# Influence of habitat structures on fish abundances and diversity: comparing mainstream and tributary communities in the urban uMsunduze Catchment, KwaZulu-Natal, South Africa

**DOI:** 10.1007/s11252-025-01688-5

**Published:** 2025-02-24

**Authors:** Nolwazi B. Ngcobo, Matthew J. Burnett, Colleen T. Downs

**Affiliations:** 1 Centre for Functional Biodiversity, School of Life Sciences, University of KwaZulu-Natal, Private Bag X01, 3209 Scottsville, Pietermaritzburg, South Africa; 2 Institute of Natural Resources NPC, P.O. Box 100 396, 3209 Scottsville, South Africa

**Keywords:** Fish communities, Habitat features, Ecological integrity, Urban

## Abstract

Various factors drive the decline of freshwater vertebrate biodiversity. These include changing landscape and urbanisation, introduced invasive species, altered habitat, water quality deterioration, instream barriers, and climate change. In the present study, we evaluated the impact of different habitat features on the fish assemblages in an urban river using catch per unit effort (CPUE) as a proxy for fish assemblage per site and season. We sampled 17 main sites and 21 ad-hoc sites in the uMsunduze Catchment in Pietermaritzburg, uMgungundlovu District, KwaZulu-Natal, South Africa, during 2022–2023. We collected data using an electro-shocker, fyke nets, and gill nets, and we also recorded and calculated habitat features such as substrate types, hydraulic biotopes, in-situ water quality, ecohydraulics, average depth, and velocity. We used Generalised Linear Models to determine the habitat features driving fish communities. We calculated the Shannon-Weiner and Pielou diversity indices to compare between rivers. We used the Fish Response Assessment Index (FRAI) tool to understand each site’s ecological integrity per season. Our results indicated that various features, including substrate (mud, sand, gravel), fast intermediate and fast deep ecohydraulics, electrical conductivity, habitat (glide, pool), and average velocity significantly impacted the CPUE of fish. There was no variation in diversity indices between tributaries, but there was a significant difference in fish diversity between the uMsunduze mainstream and its tributaries. The FRAI scores showed great deterioration in the system’s ecological health, and most sites, especially the mainstream sites, were critically or extremely modified. We suggest that the relevant authorities take action to mitigate the pressures compromising the uMsunduze Catchment’s ecological integrity. There is an urgent need for conservation measures for the two “near threatened” species, *Enteromius gurneyi* and *Amphilius natalensis*, the former now extirpated as per our study.

## Introduction

Freshwater vertebrate biodiversity is declining at a higher rate than terrestrial organisms, and multiple factors are driving these declines (Radinger et al. 2019; Tóth et al. 2019; Costa et al. 2021). Among these factors is the alteration of landscapes through land use change, such as urbanisation along rivers (Radinger et al. 2019; Tóth et al. 2019; Cruz and Pompeu 2020), the introduction of invasive species (Pool et al. 2010; Tóth et al. 2019; Lavery et al. 2022), in-stream habitat alteration and loss (Pool et al. 2010; Ramírez et al. 2012; Levin et al. 2019), water pollution (Sarkar and Islam 2020), deteriorating water quality (Ramírez et al. 2012; Mondal and Bhat 2020; Lavery et al. 2022), discharge of effluent and poorly managed wastewater (Sarkar and Islam 2020), construction of dams and impoundments (Lavery et al. 2022), climate change and changes in ambient temperature (Evans et al. 2022; Lavery et al. 2022) and changes in physico-chemical parameters (Levin et al. 2019). The survival of fish and other freshwater biota depends on mitigating these impacts to maximise the survival ability of freshwater vertebrates in highly modified freshwater environments (Gupta et al. 2012; Katopodis 2012; Reid et al. 2019).

Various environmental and spatial attributes influence fish community structures, and each plays an independent role in shaping freshwater fish meta-community assemblages (Helms et al. 2009; Gebrekiros 2016). The use of fish biological responses to understand the stability of the freshwater ecosystem has been widely adopted in aquatic ecology (Li et al. 2010; Costa et al. 2021). This is done by assessing the functional diversity within the ecosystem, for example, understanding a fish’s biological traits to its habitat preference (Li et al. 2010; Stefani et al. 2020; Shah Esmaeili et al. 2022). Functional diversity refers to the diverse traits prevailing within the ecosystem, and more functionally diverse communities tend to be more resilient to environmental stressors (Lamothe et al. 2018). With the broad responses that freshwater fish have to various anthropogenic impacts, using them to study the ecological integrity of rivers and streams has been effective (Radinger et al. 2019). Using functional diversity to evaluate prevailing functional traits in the ecosystem is a convenient approach to determining the likelihood of species persistence in the ecosystem (Shah Esmaeili et al. 2022).

Variation in flow regimes in rivers and streams is predominantly the primary driver of fish community structures and assemblages (Stocks et al. 2021). Consequently, various studies have used hydrodynamic habitat models to assess the impact of physical habitat as a flow function to determine the effect of flow variations on fish assemblages (Shukla and Bhat 2018; Wegscheider et al. 2020). Impoundment or dam introduction has a major impact on river flows, which alters the river’s hydro-morphological processes (Boavida et al. 2020; Wegscheider et al. 2020). This includes the changes in water depth, water turbulence and flow velocity, which then affect the substrate composition, hence impairing fish growth and survival (Boavida et al. 2020).

The increase in the human population is a major contribution to the alteration of land use cover and the resulting urbanisation through industrial works, deforestation, wastewater treatments, agricultural development, residential habitation and recreational grounds (Keppeler et al. 2017; Chen and Olden 2020). As a result, urbanisation has influenced over 80% of the land cover near freshwater systems (Vorosmarty et al. 2010; Arthington et al. 2016). Threatened fish species proportions are higher in areas with high human population density (Gloss et al. 2016; Chakona et al. 2022). In KwaZulu-Natal, South Africa, there have been numerous alterations in natural land cover, especially in the past 13 years because of urbanisation (Evans et al. 2022) and several threatened fish species listed as a result (Chakona et al. 2022).

The alteration of land use cover causes a decline in freshwater fish diversity (Meador et al. 2005; Levin et al. 2019). It also reduces functional diversity (Keppeler et al. 2017), supported by the decrease in the ecological integrity of rivers and streams (Levin et al. 2019), which is measured using the Fish Response Assessment Index (FRAI). This tool is widely used in South Africa to determine the ecological state of aquatic ecosystems (Kleynhans 2007; Wepener et al. 2011; Malherbe et al. 2016; Evans et al. 2022). The Fish Response Assessment Index (FRAI) calculates the ecological health of freshwater ecosystems by comparing current fish communities and habitat conditions to their natural, pristine state. The process involves conducting field surveys to collect fish species’ abundance and diversity data and assessing key habitat variables such as flow, substrate, vegetation, and water quality. These observations are then compared to reference conditions to determine deviations caused by human or environmental impacts (Kleynhans 2007; Evans et al. 2022). In the northern hemisphere, freshwater fish species have been studied and determined to be sensitive organisms to sub-catchment urbanisation (Chen and Olden 2020). Alteration in land cover features near the rivers and streams affects the physio-chemical conditions of the system (Helms et al. 2009). Physio-chemical parameters such as water temperature, pH, turbidity, total dissolved solids (TDS), dissolved oxygen (DO) and electrical conductivity (EC) influence the diversity of freshwater fish populations (Akhi et al. 2020). These are exacerbated in the urban context and can negatively impact the receiving rivers and their freshwater vertebrate populations.

In this study, we assessed various factors correlating with fish communities in the mainstream and tributaries of the uMsunduze Catchment flowing through the urbanised city of Pietermaritzburg, uMgungundlovu District, KwaZulu-Natal, South Africa. Our study aimed to quantify the functional diversity of fish species found in the mainstream and its tributaries. In addition, we assessed the fish’s biological traits and habitat associated with fish assemblages. We also investigated the differences in fish abundance and diversity within different habitat structures in the mainstream and tributaries. We predicted that fish species’ functional diversity would be higher in tributaries away from major sources of pollution than in the mainstream, which is highly fragmented and polluted.

## Methods

### Sampling sites

**Fig. 1:**
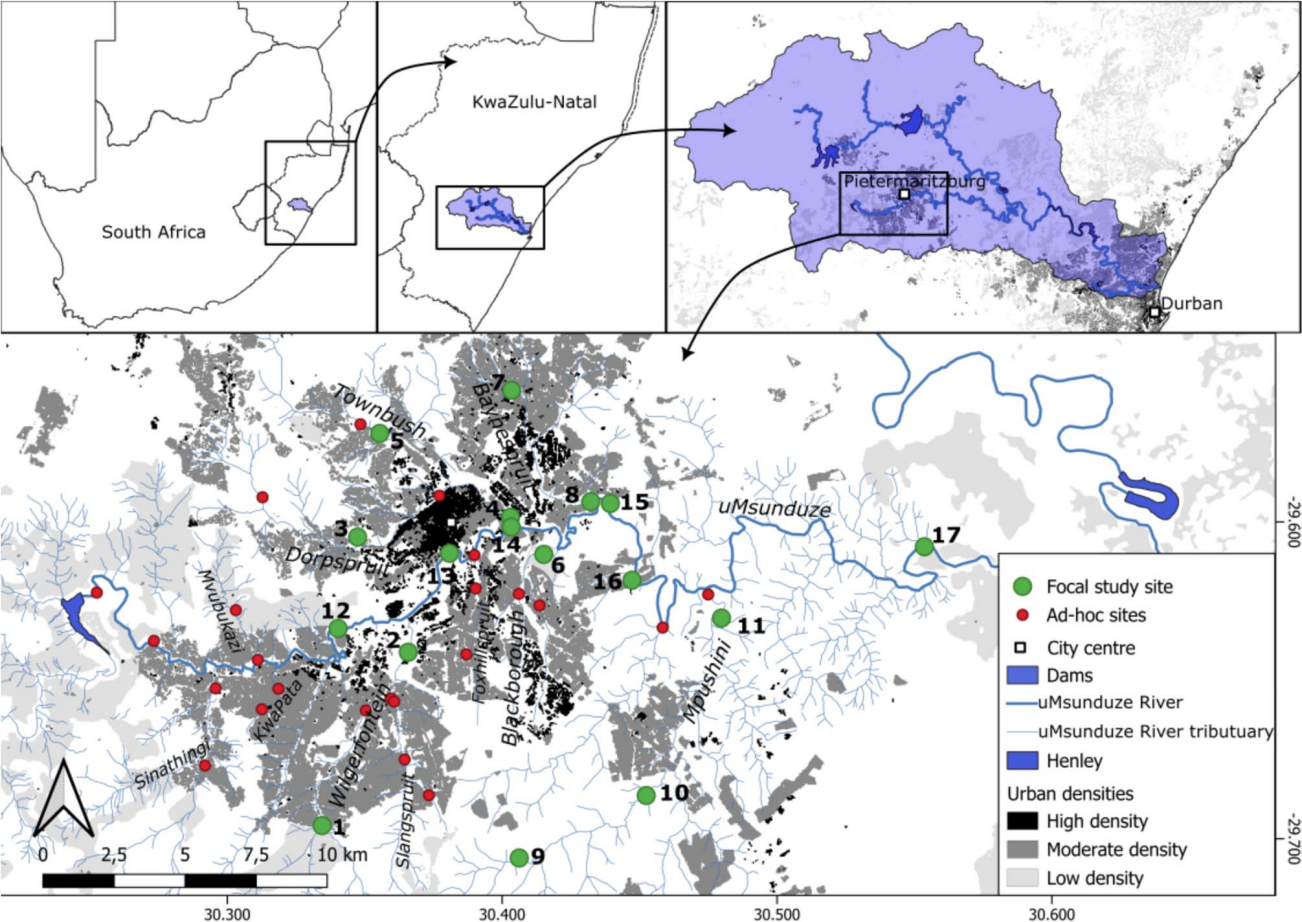
The map representation of the sites surveyed for the focal study sites and ad-hoc study sites in the uMsunduze catchment in Pietermaritzburg, umgungundlovu District, KwaZulu-Natal, South Africa

We surveyed 17 sites in seven rivers of the uMsunduze Catchment in Pietermaritzburg, uMgungundlovu District, KwaZulu-Natal, South Africa. Amongst the seven rivers, there was one mainstream and six tributaries. The area is a mosaic urban landscape (Josiah and Downs 2022), with high-density urban areas to low-density semi-rural smallholder farmers (Fig. 1). The rivers include the uMsunduze River’s mainstream, five tributaries and one sub-tributary. We selected six sites in the uMsunduze River’s mainstream, three in the Mpushini Stream, two in the Wilgerfontein, Dorpspruit and Baynespruit streams, and one in the Townbush and Blackborough streams, respectively (Fig. 1). The uMsunduze mainstream runs through peri-urban and urban areas (Henley, Imbali and Edendale) and then passes adjacent to the Pietermaritzburg city centre and continues downstream of the city where it runs through the sub-urban areas (Bishopstowe and Eastwood), followed by a wide area of agricultural land use and lastly joins the greater uMngeni River in Emvini rural area (Fig. 1).

The most upstream tributary we surveyed was the Wilgerfontein Stream, which runs through peri-urban areas of Willowfountain and Imbali suburbs. The Dorpspruit Stream was the second tributary, and its associated land use ranged from exotic tree plantations to urban residential areas (Prestbury and Winterskloof suburbs that include Wylie Park and Pietermaritzburg National Botanical Garden). In its lower reach, Dorpspruit Stream passes through the city centre, where it then joins the uMsunduze mainstream. We also surveyed one tributary of the Dorpspruit Stream, the Townbush Stream, which flows through the exotic tree plantations and urban residential areas (Cascade, Montrose and Woodlands suburbs) and joins the Dorpspruit Stream just before Pietermaritzburg’s city centre. The third uMsunduze tributary was Blackborough Stream, a reasonably short stream running through the industrial and urban areas of Mkondeni and Scottsville suburbs. The Baynespruit Stream flows through the Northdale suburbs and industrial areas. The last and most downstream tributary assessed was the Mpushini Stream, which flows through farmlands, Bisley Nature Reserve and Mpushini Conservancy and has minimal anthropogenic pressures (Fig. 1).

We had 21 additional ad-hoc sites sampled once during the study, including other minor tributaries. These were Sinathingi, Mvubukazi, KwaPata, Slangspruit and Foxhillspruit streams, which flow through moderate urban densities. The five sites off the main stem of the uMsunduze River were also included (Fig. 1).

## Sampling techniques

We used three methods for sampling fish: electro-shocking (SAMUS 725 M, Electro-fisher, SAMUS Special Electronics, Poland), which is a backpack portable electro-shocking device, and its parameters such as frequency and pulse width were adjusted based on the electrical conductivity of the water to standardise the sampling efficiency. The second method was fyke nets (2 m traps x 700 mm opening, 6 m wing and 23 mm mesh size, T&L Netmaking, Mooroolbark, Australia) and gill nets (48 mm mesh size, Eigevis group of companies, Cape Town, South Africa) in the mainstream sites that had deep pools (> 2 m deep) with one gill net per site for two sites on the mainstream. The gill nets were installed and soaked for 2 h in each site. All sites were sampled using the electro-shocking technique. We placed temporary barriers in small streams by running seine nets (2 mm mesh size, 50 cm height) upstream and downstream of the 30 m sampled area. We did two passes with the electro-shocking, which constituted an effort for each pass with the time taken for each effort recorded. In addition, two fyke nets were deployed for 2 h only at the Mpushini main sites and Dorpspruit upstream site as habitat for this method was available (Fig. 1). We conducted our surveys quarterly in March, June, August and November of 2022 to factor in the seasonal factor in the fish assemblages; with the sampling conducted once in each site per quarter. To fill in the gaps, we sampled ad-hoc sites during January 2023 to coincide with high flows when the presence of fish in the tributaries would be greatest. The ad-hoc sites were selected between the focal study sites to identify any gaps in the spatial distribution of fish species. All caught fish were handled as per Evans et al. (2022). We used Skelton (2001) to identify fish to species. The standard length was measured for all fish collected, and the total per species was recorded for each site together with the sampling effort. We then returned and released all the fish to the river where we had caught them (see details in Ngcobo (2024) and Ngozi (2024).

At all the main sites and where we caught fish at the ad-hoc sites, the habitat was recorded, including the total length of the surveyed river stretch and the cross-section every five meters. For each cross-section, five points were collected perpendicular to the flow of the river as per Ellender et al. (2012), with depth, velocity, substrate, and respective biotopes recorded. For velocity and depth, the transparent velocity head rod (TVHR, Groundtruth, Hilton, South Africa) was used (Desai et al. 2021; Evans et al. 2022). The substrate types recorded were bedrock, boulders, cobbles, gravel, sand, and mud. We recorded the hydraulic biotopes, such as pool, still margin, glide, run and rapid, as defined by Wadeson (1995). We converted depth and velocity readings per point into eco-hydraulic classes as per James and King (2010). The ecohydraulics were classified into seven classes: fast very shallow (FVS), fast shallow (FS), fast intermediate (FI), fast deep (FD), slow very shallow (SVS), slow shallow (SS) and slow deep (SD) (James and King 2010).

## Data analyses

We calculated the catch per unit effort (CPUE) as a density estimate for fish per m^3^, using the following formula:

$$\:CPUE=\frac{\sum\:({x}_{1}+{x}_{2}+{x}_{3}+\dots\:{x}_{\infty\:})}{\sum\:(L\times\:B\times\:H)}$$

Where X_1−*∞*_ represents the number of individuals per species. L x B x H is a calculation for the area per cross-section where L represents the length of along the riverbank, B represents the width of the respective cross-section, and H stands for average depth in each cross-section. Therefore, the CPUE is presented by the relative abundance of fish per area surveyed in each quarter sampled (Ellender et al. 2012). The CPUE was then used as a dependent variable in the analyses. For the independent variable, we converted the habitat variables to proportions compared with other variables in similar categories.

We used analysis of variance (ANOVA) multivariate statistical analysis performed in R (version 4.2.2) to determine which habitat features significantly impacted the fish community structures within the catchment. To evaluate the effects of habitat features, we ran many generalised linear models (GLMs) using the mvabund package (Desai et al. 2021). For the GLMs, CPUE was used as a function of each factor within the habitat features. We ran several models where CPUE was a function of substrate (mud, sand, gravel, cobbles, boulders and bedrock), biotopes (still margin, pool, glide, run and rapids), in-situ water quality (water temperature, pH, electric conductivity, salinity and total dissolved solvents), ecohydraulics (FVS, FS, FI, FD, SVS, SS, SD), average depth and velocity, and barrier density per kilometre.

To measure the diversity and evenness of fish species communities per river, we calculated the Shannon-Weiner diversity index (H’) and the Pielou’s evenness index (J’). The Shannon-Weiner index was calculated from the species abundance data using vegan packages in R (v.4.2.2). The Pielou’s evenness is the natural logarithm of the Shannon index {(ln (H’)}. We conducted a Kruskal-Wallis test to determine if there was a significant difference in species diversity between rivers.

Lastly, to determine the ecological class for each site per season, we used the Fish Response Assessment Index (FRAI) (Kleynhans 2007), a tool designed to quantify the ecological integrity of the river site associated with monitoring for excessive water resource use with the South African National Department of Water and Sanitation. This tool uses historical data on species distribution and their intolerance of environmental changes in habitat features over time. Individual components are scored and weighted based on their ecological importance, and the results are integrated to produce a final FRAI score, expressed as a proportion (0–100). We obtained historical fish species distribution from the Freshwater Biodiversity Information System (FBIS n.d.), and for all our sites, we used the uMsunduze Catchment species distribution. The FRAI tool gives the results as adjusted and general ecological scores and classes to describe the state of the river (Table 1).

**Table 1:** The ecological classes, scores and description of ecological integrity as depicted by Kleynhans and Louw (2007)

Ecological Class	Name	Ecological Score	Description
A	Natural	90–100	Natural
B	Good	80–89	Mostly natural but with light modification
C	Fair	60–79	Moderately modified
D	Poor	40–59	Largely modified
E	Seriously Modified	20–39	Seriously modified
F	Critically Modified	0–19	Extremely modified

## Results

**Fig. 2:**
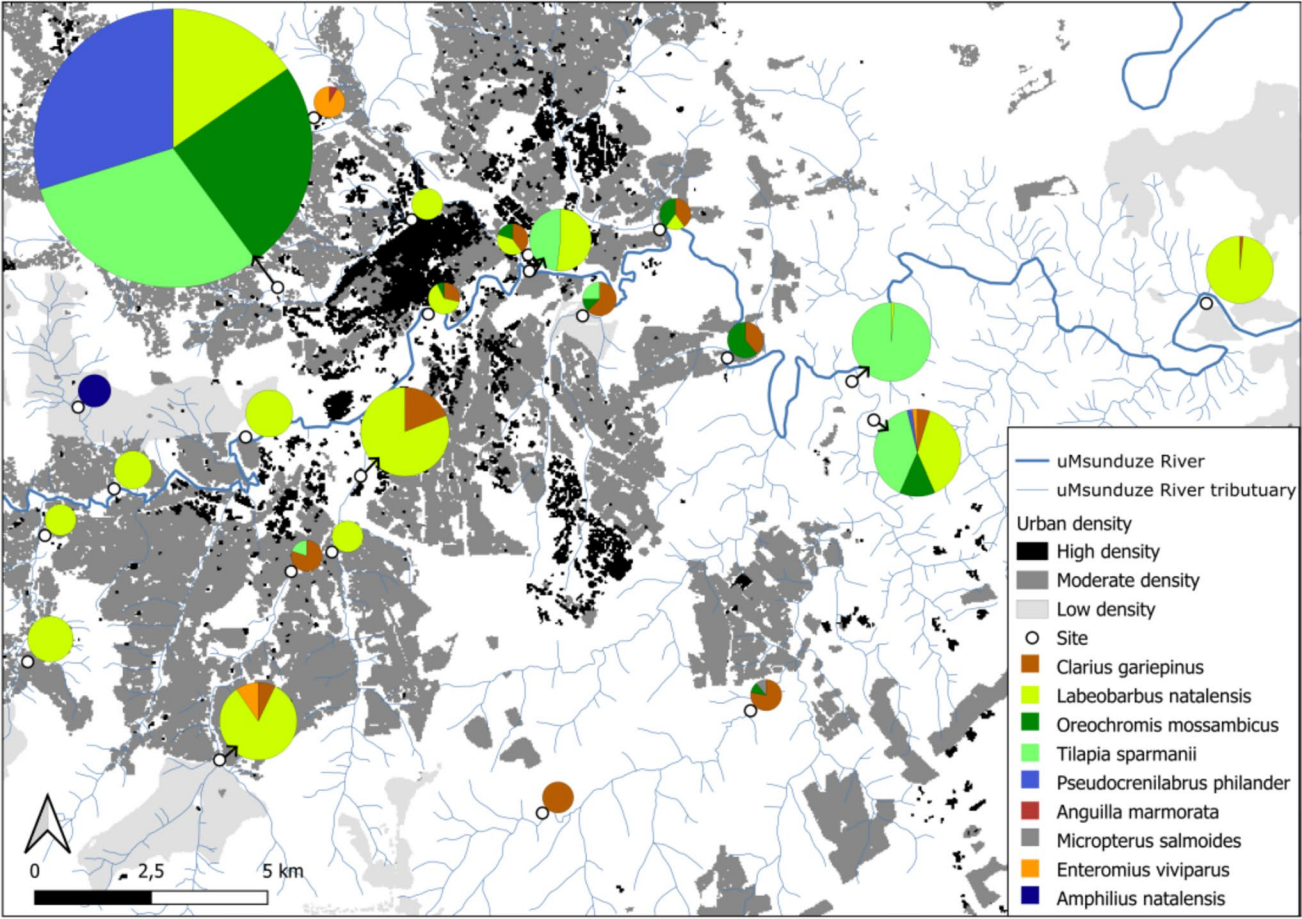
Spatial representation of fish species abundances on different rivers and sites in the uMsunduze Catchment, Pietermaritzburg, uMgungundlovu District, KwaZulu-Natal, South Africa, for the main and ad-hoc surveys- Pie charts representing species diversity per site

A total of 1798 fish from nine species and seven families were caught during the study. The most common species we caught during our study were *Labeobarbus natalensis* (*n* = 573), *Tilapia sparmanii* (*n* = 489), *Pseudocrenilabrus philander* (*n* = 326), *Oreochromis mossambicus* (*n* = 299), and *Clarius gariepinus* (*n* = 73). The abundances of these species were highest at the upper Dorpspruit (3) sites (Fig. 2; Table 2). The other species were only caught once or relatively few times and in smaller abundance. These included *Amphilius natalensis* (*n* = 15) caught during the ad-hoc survey at the upstream Mvubukazi (20) site (Fig. 2; Table 2). *Anguilla mossambica* (*n* = 1) and *Micropterus salmoides* (*n* = 1) had only one individual of each caught once at the Townbush (5) and middle Mpushini (10) sites respectively (Fig. 2; Table 2). *Enteromius viviparus* (*n* = 21) was caught at three localities in the upper Wilgerfontein (1), Townbush (5) and downstream Mpushini (11) sites (Fig. 2; Table 2).

**Table 2:** List of expected and observed species and their IUCN listing, as outlined by Chakona et al. (2022), Evans et al. (2022) and freshwater biodiversity information system website for native species and IUCN for alien species, in the uMsunduze Catchment in Pietermaritzburg, uMgungundlovu District, KwaZulu-Natal, South Africa

Expected Species	Native/Invasive		Historical presence	Current occurrence	IUCN listing
*Amphilius natalensis*		Native	X	X	NT
*Anguilla bengalensis*		Native	X		LC
*Anguilla mossambica*		Native	X	X	LC
*Carassius auratus*		Alien	X		LC
*Clarius gariepinus*		Native	X	X	LC
*Cyprinus carpio*		Alien	X		LC
*Enteromius gurneyi*		Native	X		NT
*Enteromius toppini*		Native	X		NT
*Enteromius viviparus*		Native	X	X	NT
*Labeo molybdinus*		Native	X		LC
*Labeobarbus natalensis*		Native	X	X	LC
*Lepomis macrochirus*		Alien	X		LC
*Micropterus punctulatus*		Alien	X		LC
*Micropterus salmoides*		Alien	X	X	LC
*Oreochromis mossambicus*		Native	X	X	VU
*Pseudocrenilabrus philander*		Native	X	X	LC
*Tilapia sparmanii*		Native	X	X	LC

*Enteromius gurneyi* was not found during the study, and *Amphilius natalensis* was only caught once during the ad-hoc survey (Table 2; Fig. 2). We expected to find 18 species in the uMsunduze Catchment using the Freshwater Biodiversity Information System, but we only caught 50% of these species in the present study (Table 2; Fig. 2).

**Fig. 3:**
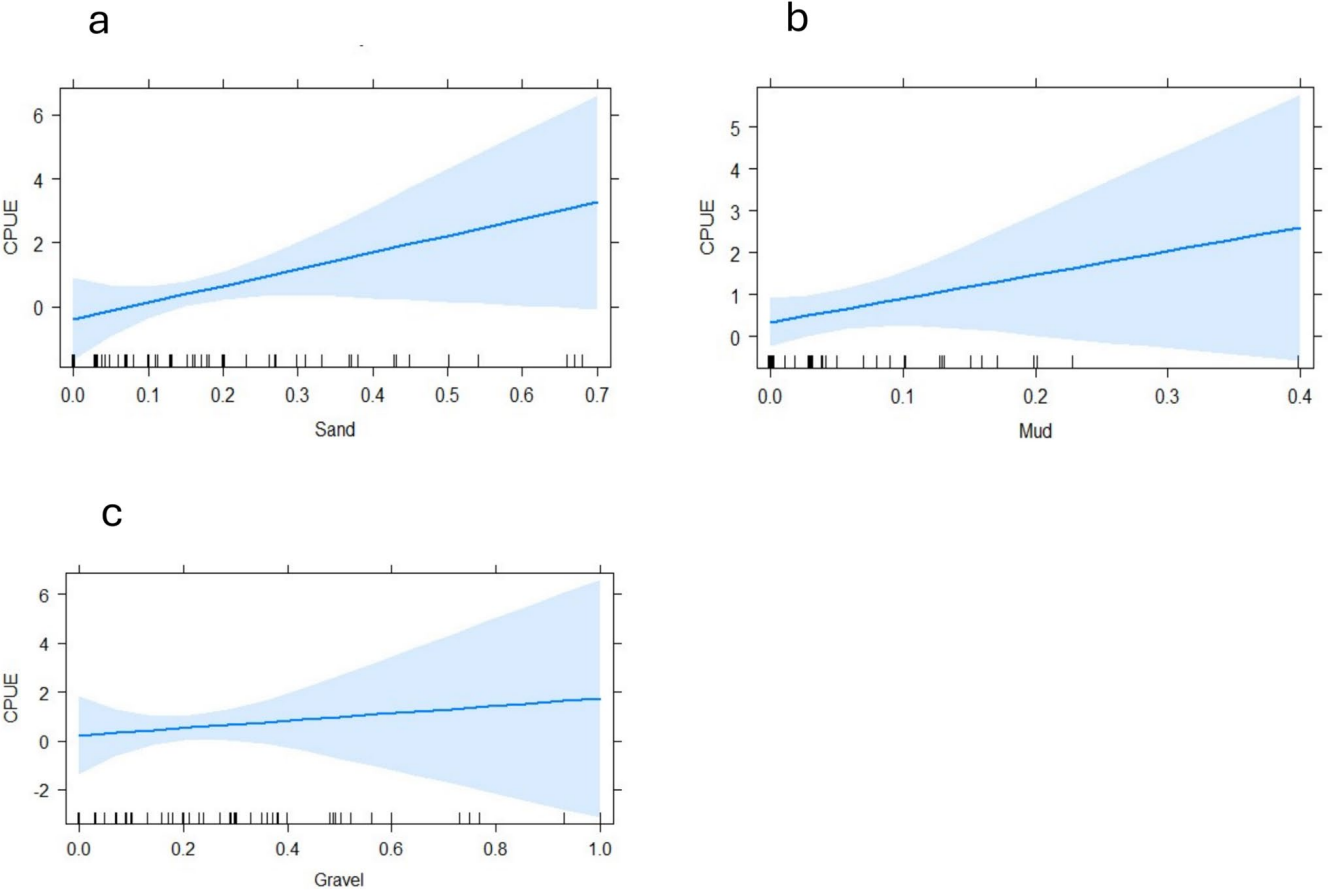
Generalised linear model outputs presenting the significant factors in catch per unit effort (CPUE) of fish for substrate types (**a**) sand, (**b**) mud and (**c**) gravel in the uMsunduze Catchment in Pietermaritzburg, uMgungundlovu District, KwaZulu-Natal, South Africa

**Fig. 4:**
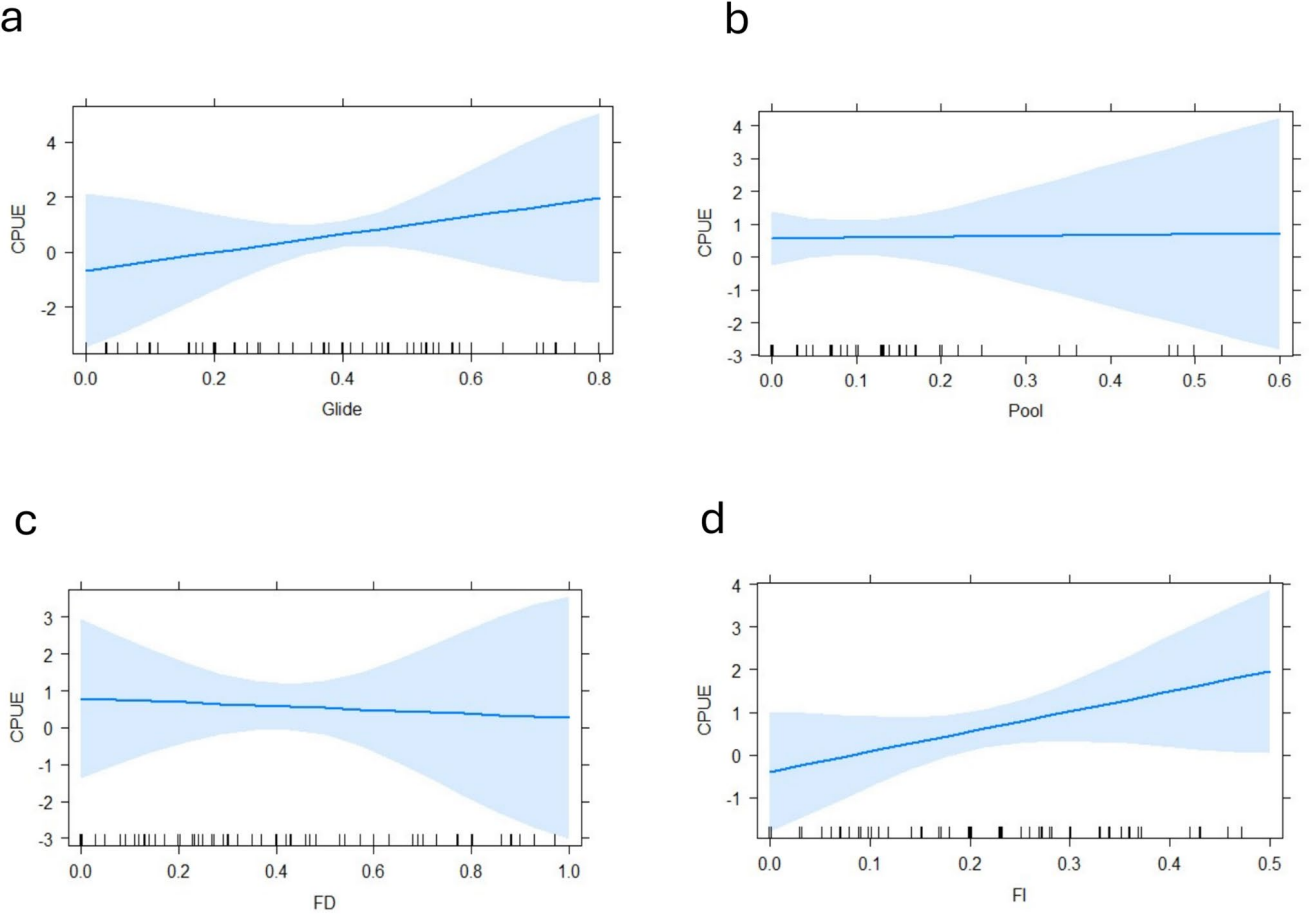
The generalised linear model outputs depicting the significance of glide and pool factors among the biotopes’ habitat features where (**a**) is glide, and (**b**) pool, and showing the significant attributes of ecohydraulics on the catch per unit effort of fish where (**c**) shows fast deep (FD), and (**d**) fast intermediate (FI), in the uMsunduze Catchment in Pietermaritzburg, uMgungundlovu District, KwaZulu-Natal, South Africa

**Fig. 5:**
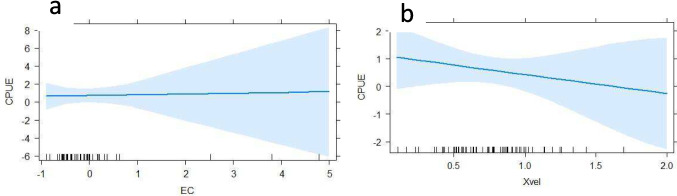
Generalised linear model output showing (**a**) the significance of transformed electric conductivity (EC) on the catch per unit effort of fish under the in-situ water quality parameters, and (**b**) the impact of water velocity (Xvel) on the catch per unit effort (CPUE), in the uMsunduze Catchment in Pietermaritzburg, uMgungundlovu District, KwaZulu-Natal, South Africa

**Fig. 6:**
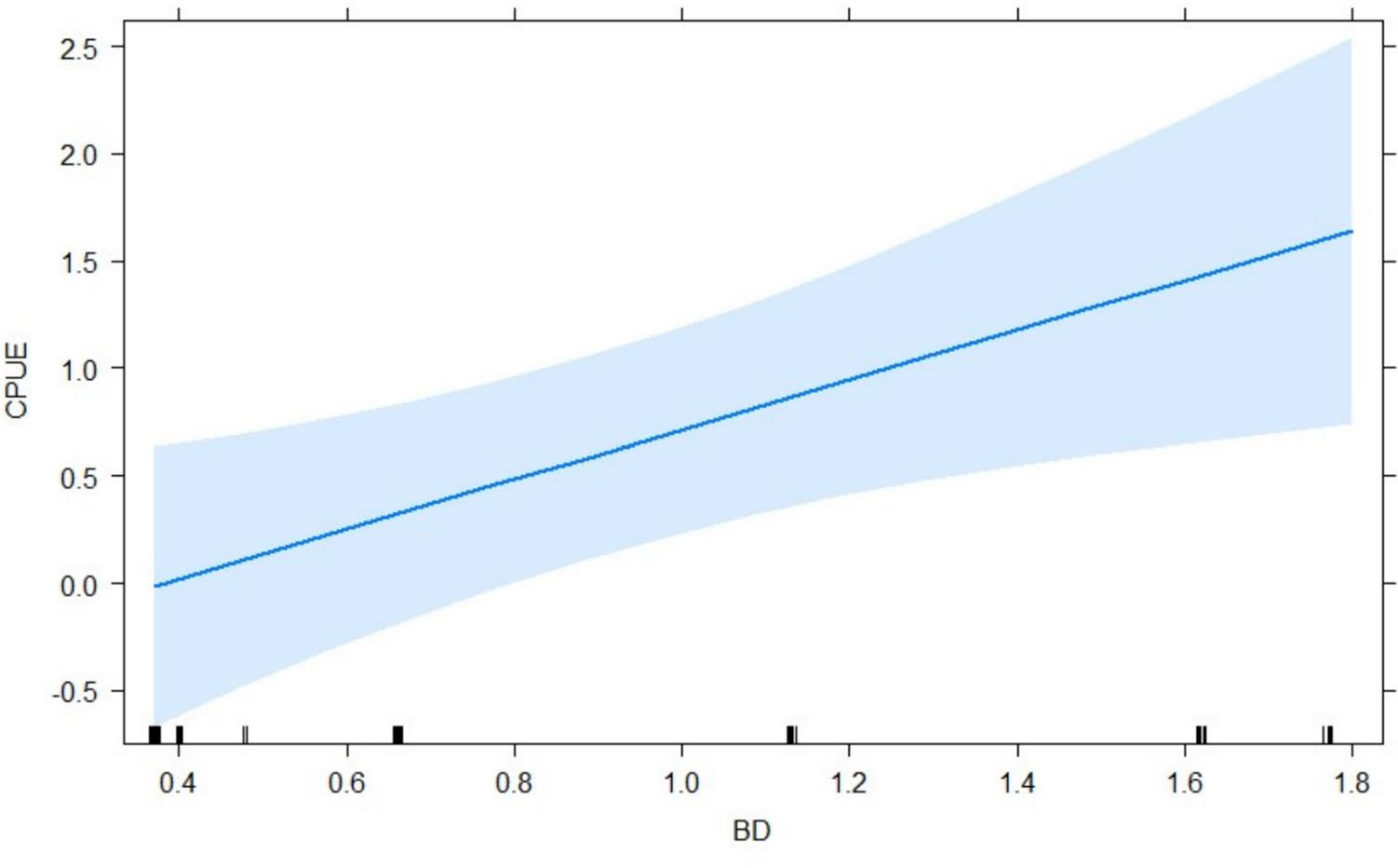
Generalised linear model showing the significance of catch per unit effort (CPUE) response to physical barrier density/km (BD) in the uMsunduze Catchment in Pietermaritzburg, uMgungundlovu district, KwaZulu-Natal, South Africa

The habitat variables significantly affected CPUE (ANOVA, F = 3.966; DF = 4 ; *P* = 0.029). Substrate variables included mud (*P* = 0.003), sand (*P* = 0.001) and gravel (*P* = 0.017) (Fig. 3). The biotopes glide (*P* = 0.001) and pool (*P* = 0.027) significantly impacted the CPUE (Fig. 4). Most ecohydraulics did not show significance, but fast deep (*P* = 0.003) and fast intermediate (*P* = 0.035) were significant (Fig. 4). Only conductivity was a significant in-situ water quality variable (*P* = 0.045) (Fig. 5). Average velocity significantly impacted the CPUE (*P* = 0.033) (Fig. 5). Lastly, the barrier density per kilometre significantly impacted the CPUE such that CPUE increased as the barrier density increased (Fig. 6).

**Fig. 7:**
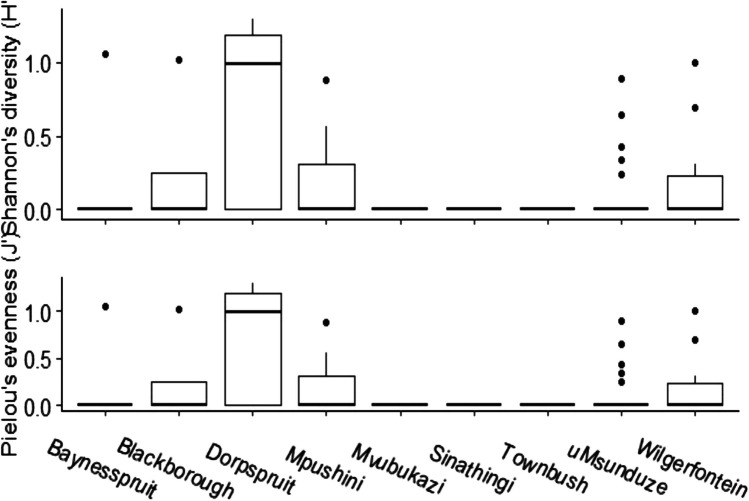
The Shannon-weiner diversity index (H’) and pielou evenness index (J’) for the focal study tributaries, ad-hoc surveys, and the uMsunduze River mainstream

Both the Shannon-Weiner and Pielou evenness diversity indices for fish communities were variable (Fig. 7). There were no significant differences between the rivers; Shannon diversity ‘H’ (Kruskal-Wallis Chi-squared = 10.535, df = 8, *P* > 0.2295) and Pielou evenness J’ (Kruskal-Wallis Chi-squared = 10.535, df = 8, *P* > 0.2295). However, there was a significant difference between the uMsunduze and Dorpspruit diversity indices (Pairwise comparison Wilcoxon rank, *P* = 0.024). The uMsunduze mainstream did not show significant diversity in both Shannon-Weiner and Pielou diversity indices (Fig. 7). In contrast, there was relatively high diversity in the tributaries. Dorpspruit showed the highest species diversity, followed by Mpushini, Blackborough and Wilgerfontein streams, while all other tributaries showed no significant diversity (Fig. 7). Our results showed that the sites with the highest diversity and evenness also had the highest fish abundances (Figs. 2 and 7).

**Fig. 8:**
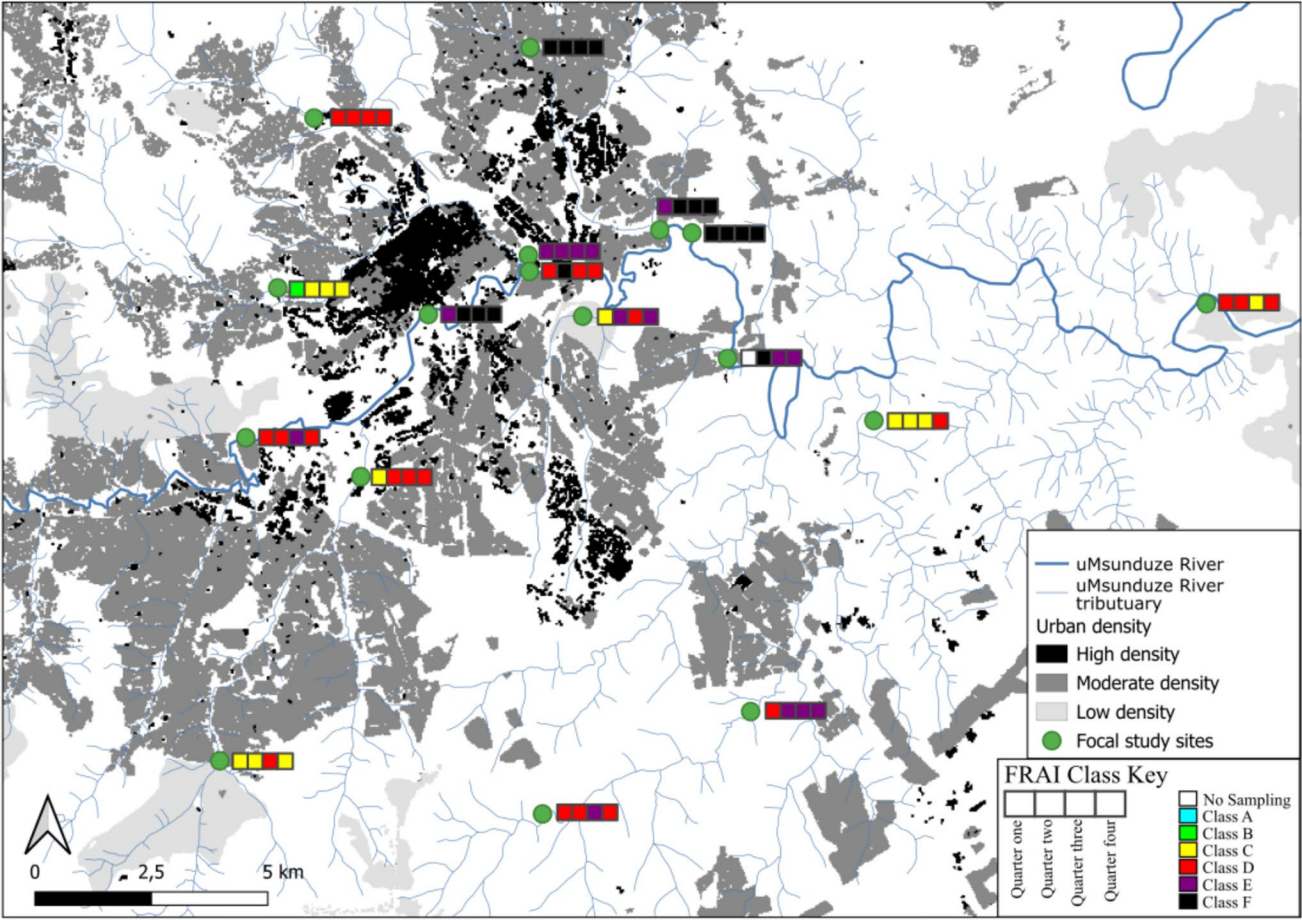
The adjusted FRAI classes for the main study sites for four surveys conducted seasonally in the uMsunduze Catchment in Pietermaritzburg, uMgungundlovu District, KwaZulu-Natal, South Africa

Most of our sampling sites had a declining trend using the FRAI scores across the different quarters (Table 3; Fig. 8). Our FRAI scores suggest that seasonal changes put increased pressure on the system (Table 3; Fig. 8). Most sites in the mainstream were either “Largely Modified”, “Seriously Modified”, or “Extremely modified” throughout the study period (Table 3; Fig. 8). We did not get scores that suggest that any site was in its natural or pristine state (Table 3; Fig. 8). We did, however, have one site (site three, Dorpspruit upstream) that was “Mostly natural but with light modification”; this was for one season, and the site was in a “moderately modified” state after that (Tables 1 and 3).

**Table 3:** The seasonal changes in the fish response assessment index (FRAI) ecological scores for each site over the four quarters sampled in the uMsunduze Catchment in Pietermaritzburg, uMgungundlovu District, KwaZulu-Natal, South Africa. (see Fig. 1 for localities)

		Seasonal Quarters			
River/Stream	Site no.	March	June	August	November
Wilgerfontein	1	63.2	63.6	56.7	62
Wilgerfontein	2	64.8	56.8	48.6	53.4
Dorpspruit	3	82.7	68.8	70.4	71.2
Dorpspruit	4	35.2	34.5	36	36
Townbush	5	57.6	54.4	49	51.9
Blackborough	6	64.8	37.3	42.4	37.8
Baynespruit	7	16.3	16	15.7	16.2
Baynespruit	8	24.6	18.1	18.6	18.3
Mpushini	9	55	53.1	54	54.5
Mpushini	10	52	48.3	50.6	51.3
Mpushini	11	63.6	63.2	56.8	57
uMsunduze	12	57	56	54.2	57.8
uMsunduze	13	28	19	18.3	18.4
uMsunduze	14	48	46.3	51.8	50.6
uMsunduze	15	18.7	18.3	17.8	17.5
uMsunduze	16	N/A	18.3	22.3	26.4
uMsunduze	17	55.8	52	66.1	57.3

## Discussion

Our study on the uMsunduze Catchment in Pietermaritzburg, KwaZulu-Natal Province, found that the overall fish diversity consisted of nine species, 50% of the 18 expected species. This was relatively poor compared with the 64% of KwaZulu-Natal indigenous species found in KwaZulu-Natal major rivers in 2016 (Evans et al. 2022). The uMsunduze mainstream showed no significant differences in diversity when comparing the Shannon-Weiner and Pielou diversity indices. As we predicted, the tributaries had relatively higher diversity, with Dorpspruit showing the highest species diversity and abundance. The sites with the highest diversity and evenness also had the highest fish abundance. However, four out of nine species were found in low abundance, which suggests that these species are also at risk of extirpation from the study area.

The uMsunduze Catchment is a highly polluted river (pers. obs., Gemmell and Schmidt 2013; Ngcobo 2024), and there have been various major fish kills in the past (Karssing 2007; Ngozi 2024). This pollution could significantly decrease fish diversity and abundance in the system (Evans et al. 2022). Two of the native species, *A. natalensis* and *E. gurneyi* are listed as “Near threatened” by the IUCN Red List (Chakona et al. 2022). We did not detect *E. gurneyi*, and *A. natalensis* was only caught once during an ad-hoc survey. *Enteromius gurneyi* had a type specimen collected near the uMsunduze (16) site confirming historical records for the species (O’Brien et al. 2017), which calls for conservation measures that need to be taken for the species.

We found that most of our sampling sites had a declining trend using the FRAI scores. The results from our FRAI scores suggest that seasonal changes impact fish communities on the system, with decreased scores in low flow conditions. Most sites in the mainstream were either a Class D “Largely Modified”, Class E “Seriously Modified”, or Class F “Extremely modified” throughout the study. Across the study area, we did not get scores suggesting that these streams are in a natural or pristine state. We did, however, have one site that was a Class B “Good” the upper Dorpspruit (3) site; this was for one season only, during a high flow period. Generally, a natural ecological FRAI class would indicate that the ecosystem can support and uphold a cohesive, adaptable community of organisms, and it can exhibit species composition, diversity, and functional diversity comparable to that found in pristine habitats of a particular region (Kwak and Freeman 2010). Despite this score, key species, such as *E. gurneyi*, *A. natalensis*, and the *Anguillid* spp., were still missing, indicating that this site may not assist in conserving these key species (IUCN Red listing 2016; Chakona et al. 2022).

Most of our sites were in or near urban built areas or high-density building areas, including the city centre. Urbanisation is one factor that has been documented to impact the functioning of freshwater ecosystems (Evans et al. 2022). The FRAI scores indicated there was a seasonal change in fish community assemblages, and the upstream Dorpspruit (3) site decreased from Class B, “good” to Class C “moderately modified” state, which showed that the wetter warmer season (austral Summer) improved ecological scores for fish and drier cooler season (austral winter) reduced ecological scores. Although the upper Dorpsruit (3) site had the highest abundance and diversity per site, it only had four of the 18 expected fish species. The downstream of the same stream was at a Class D “seriously modified” state throughout all seasons; similar results were seen in Vaal River, Gauteng, South Africa (Wepener et al. 2011). The ecological scores indicated that high urban density areas compromised the ecological integrity of the ecosystem; in essence, the upstream was not as impacted as the midstream and downstream sections. Similarly, in the Nakdong River, South Korea, the ecological state showed that the river was highly impaired from the middle to downstream after the impact of industrial land use and wastewater treatment (Kim and An 2015). Overall, the ecological state of the uMsunduze River is poor, with concerns for worsening in areas with higher human disturbances. Furthermore, there is a need for immediate management and maintenance actions, such as controlled disposal of waste into the river (Hara et al. 2019) and mitigation against *E. coli* inputs (Gemmel and Schmidt 2013).

Our study’s most significant habitat features were substrate features, sand, gravel and mud. The two common species we sampled, *C. gariepinus* and *O. mossambicus*, prefer muddy substrates (Skelton 2001), and support the significance of muddy substrates on the catch per unit effort. The significant abundance of species like *C.* *gariepinus* and *O. mossambicus* in muddy substrates highlights the role of substrate preferences in shaping functional traits, particularly for species tolerant of diverse environmental conditions, including pollution and high turbidity (Skelton 2001; Russell et al. 2012). *Oreochromis mossambicus* has been previously noted to be tolerant of a wide range of physico-chemical pollution, for example, ammonium nitrogen in human effluents, acidic and alkaline environments, high altitude impoundments and high turbidities (Russell et al. 2012). The significance of gravel can be explained by the high gradient of the region’s rivers (Ngcobo 2024), which also supports the relatively high abundance of *L. natalensis* caught in the study. Female *L. natalensis* prefer gravel beds for spawning, and the offspring are associated with gravel before they can move freely between habitats (Karssing 2007; Dlamini 2019). This species also tends to occur in shoals, and it would be expected to catch them in higher abundance when present (Karssing 2007; Burnett et al. 2021). Due to their rheophilic nature, *L. natalensis* occupies both fast and slow-moving waters during summer and winter, respectively (Burnett et al. 2021); hence, the decrease in catch per unit effort when the average velocity increased could be explained by the seasonal movements of *L. natalensis* associated with different habitats in the present study. Furthermore, *O. mossambicus*, which was dominant in the system, prefers quiet open pools with slow-moving waters (Jayaratne and Surasinghe 2010; Russell et al. 2012). Cichlid species have various substrate preferences, including accumulating nesting “beds” in sandy and muddy substrates (Dario et al. 2018), where they breed in low or no flows (Mckaye 1977). For instance, Dlamini (2019) and Evans et al. (2022) found that *P. philander* and *O. mossambicus* were associated with sandy substrates. In our study, we caught three species that belonged to the Cichlidae family: *O. mossambicus*, *P. philander* and *T. sparmanii*, which were both relatively common at certain sites and were usually found in higher abundance, hence the significance of sand and mud on the catch per unit effort (Wasserman et al. 2016). Cichlidae species have also been noted to prefer sandy, rocky substrates, and in this case, it would explain the significance of gravel and sand substrates because all cichlids were found in higher abundance as in other studies (Kinyage and Lamtane 2018). In particular, the high abundance of cichlids in the present study was in the upper Dorpspruit (3) site, where there is a weir pool that has sandy/mud substrates and low flows, ideal for cichlids.

According to our results, there was an increase in the availability of glide biotopes, resulting in higher catch per unit effort, while the more pools present caused a decrease. During spawning, *L. natalensis* migrate to glides to spawn in the gravel associated with the glides (Karssing 2007; Dlamini 2019). We had higher abundances of *L. natalensis* than any other species; hence, the significance of the habitat features could be influenced by the species that were found in greater abundance.

Fast deep and fast intermediate ecohydraulic types significantly impacted the catch per unit effort in our study. Fast intermediate ecohydraulics resulted in a higher catch per unit effort, while fast deep resulted in a slight decrease. However, the increase in velocity was associated with decreasing catch per unit effort. Some species we found, like *L. natalensis*,* A. natalensis*, and juveniles of *A. mossambica* and *C. gariepinus*, are semi-rheophilic species, hence the significance of fast-flowing waters and slow-moving waters or glides for differing biological needs (Burnett et al. 2021; Evans et al. 2022). *Tilapia sparmanii* prefers shallow habitats with vegetation (Wasserman et al. 2016; Evans et al. 2022); hence, deep habitat cover decreased the catch per unit effort. For the *Anguilla* species, *A. bengalensis* is more unlikely to occur in the study area (Hanzen et al. 2020), although instream barriers have impacted both species’ distribution on the uMngeni River and they now occur in low abundance (Hanzen et al. 2022). This could explain the low abundance of Anguillids despite their tolerance for poor water quality (Hanzen et al. 2022). This is supported by a 47% decline in *A. mossambica* between 1985 and 2020 in South Africa (Hanzen et al. 2022).

Within the physicochemical parameters, electric conductivity was the only significant variable affecting catch per unit effort. As the electrical conductivity increased, there was a slight increase in the catch per unit effort. Similarly, Mueller et al. (2017) found a positive correlation between electrical conductivity and catch per unit effort. In some species, like *C. gariepinus*, water parameters do not play a significant role, possibly because this species is tolerant to a wide range of environmental conditions (Karssing 2007; Evans et al. 2022). The significance of the high barrier density can be explained by the fact that species cannot move between fragments; in essence, the Dorpspruit and Wilgerfontein streams had the highest barrier density (Ngcobo 2024), and they also had the highest fish species abundance. In previous studies, fish populations have modified their life-history strategy to enhance residency if they fail to migrate across the barriers (Branco et al. 2017). This could be particularly true for potamodromous species that can still access their required habitats within the newly fragmented river reach; however, this may reduce genetic resilience in the long term (O’Brien et al. 2019; Burnett et al. 2021). A river needs to be considered in urban river linear infrastructure management to maintain connectivity between the river’s mainstream and its tributaries to improve the resilience of fishes faced with the plethora of urban stressors as found in the present study (Mantel et al. 2017; O’Brien et al. 2019).

The dominance of *O. mossambicus* and other cichlids, such as *P. philander* and *T. sparmanii*, further emphasises the importance of sandy and muddy substrates for breeding and habitat use. The adaptability of cichlids to various substrate types and their tendency to form nesting beds highlights their ecological versatility, contributing to functional diversity within the system. Similarly, ecohydraulic types such as fast intermediate flows supported semi-rheophilic species, while slower-moving habitats like glides provided critical spawning grounds for *L. natalensis.*

## Conclusions

Our study showed that fish communities in the uMsunduze Catchment are in relatively poor condition and need intervention to improve species presence, abundance and diversity. Species like *A. natalensis* are rare in the system, and the declines are supported by the deterioration in the ecological integrity of the streams (Evans et al. 2022), and our study supports its listing as “near threatened” IUCN status. The IUCN “vulnerable” listed *E. gurneyi* species was not found in the uMsunduze Catchment despite the effort of the ad-hoc surveys; this species can no longer be reliably found in the system and is likely extirpated from the study area. The poor ecological state of the uMsunduze mainstream jeopardises the potential for a recovery of the threatened species. Further, the uMsunduze River’s tributaries were in better ecological states than the mainstream and potentially can be used as refugia for species less tolerant to pollutants. However, this is only true for some tributaries. For example, the Mvubukazi Stream was the only site to have *A. natalensis*. Hence, it is important that care be taken of these tributaries and efforts made to mitigate and restore the uMsunduze River’s ecological state to protect freshwater vertebrate species. This can be done by mitigating factors driving the decline of fish species and other freshwater taxa, like reducing industrial and domestic pollution and improving wastewater treatment and effluent.

## Data Availability

The data belong to the University of KwaZulu-Natal and are available from the authors on reasonable request.
